# Primary Tracheal Schwannoma Presenting With Acute Airway Compromise: A Rare Cause of Rapidly Progressive Breathlessness

**DOI:** 10.7759/cureus.113125

**Published:** 2026-07-21

**Authors:** Kanika Balasubramani, Kanishthica Vellaichamy Ramesh Prabu, Mohammed Siddique, Vikram V. J., Swaminathan Kalyanasundaram

**Affiliations:** 1 Medicine, Madras Medical College and Rajiv Gandhi Government General Hospital, Chennai, IND; 2 Otolaryngology - Head and Neck Surgery, Madras Medical College and Rajiv Gandhi Government General Hospital, Chennai, IND; 3 Pathology, Madras Medical College and Rajiv Gandhi Government General Hospital, Chennai, IND

**Keywords:** airway obstruction, benign tracheal neoplasm, emergency tracheostomy, intratracheal tumor, tracheal schwannoma

## Abstract

Schwannoma is a benign neoplasm arising from Schwann cells and commonly affects peripheral nerves in the head and neck region. Primary intratracheal schwannoma is exceedingly rare and may present with nonspecific respiratory symptoms, often leading to diagnostic delay. We report the case of a 21-year-old male patient who presented with a two-month history of cough with expectoration and progressively worsening breathlessness for two weeks. The patient developed acute respiratory distress necessitating emergency tracheostomy. Contrast-enhanced computed tomography demonstrated a well-defined intraluminal tracheal mass causing critical airway narrowing. Bronchoscopic evaluation confirmed a smooth obstructive lesion. Histopathological examination revealed spindle-shaped cells arranged in Antoni A and Antoni B patterns, with strong diffuse S-100 protein positivity on immunohistochemistry, confirming the diagnosis of schwannoma. Primary intratracheal schwannoma, though rare, should be considered in young patients presenting with unexplained and rapidly progressive airway obstruction. Early imaging and bronchoscopic evaluation are crucial for timely diagnosis. Prompt surgical intervention is potentially life-saving in cases presenting with acute airway compromise.

## Introduction

Schwannomas are benign (non-cancerous) tumors (neoplasms) that grow from Schwann cells, which form the protective outer layer (peripheral nerve sheath) of our nerves. While these tumors can develop along almost any nerve in the body, they rarely affect the nerves responsible for smell (olfactory) and sight (optic) because those nerves lack a Schwann cell covering. A large portion of these tumors occur in the head and neck. However, primary involvement of the trachea (the windpipe) is exceedingly rare, making up less than 0.5% of all tumors found in the airway. When they do occur in the respiratory system, they are more commonly found deeper inside the lungs (distal tracheobronchial tree, including the bronchi and lung tissue) [1]. In contrast, the most common cancerous (malignant) tumors of the windpipe are squamous cell carcinoma and adenoid cystic carcinoma [2].

​Here, we present a rare case of a primary schwannoma growing inside the windpipe (intratracheal) of a young adult. The tumor physically blocked the airway, causing rapidly worsening breathing difficulties (progressive respiratory distress) that required an emergency surgical opening in the neck to restore breathing (tracheostomy).

## Case presentation

A 21-year-old male patient presented with a two-month history of a persistent cough productive of mucoid, non-foul-smelling expectoration, for which he had been taking over-the-counter medications. He reported approximately 10 episodes of hemoptysis over the preceding month but denied any voice changes or noisy breathing (stridor) during this initial period. There was no history of fever, weight loss, smoking, or prior pulmonary disease, including asthma.

**Figure 1:**
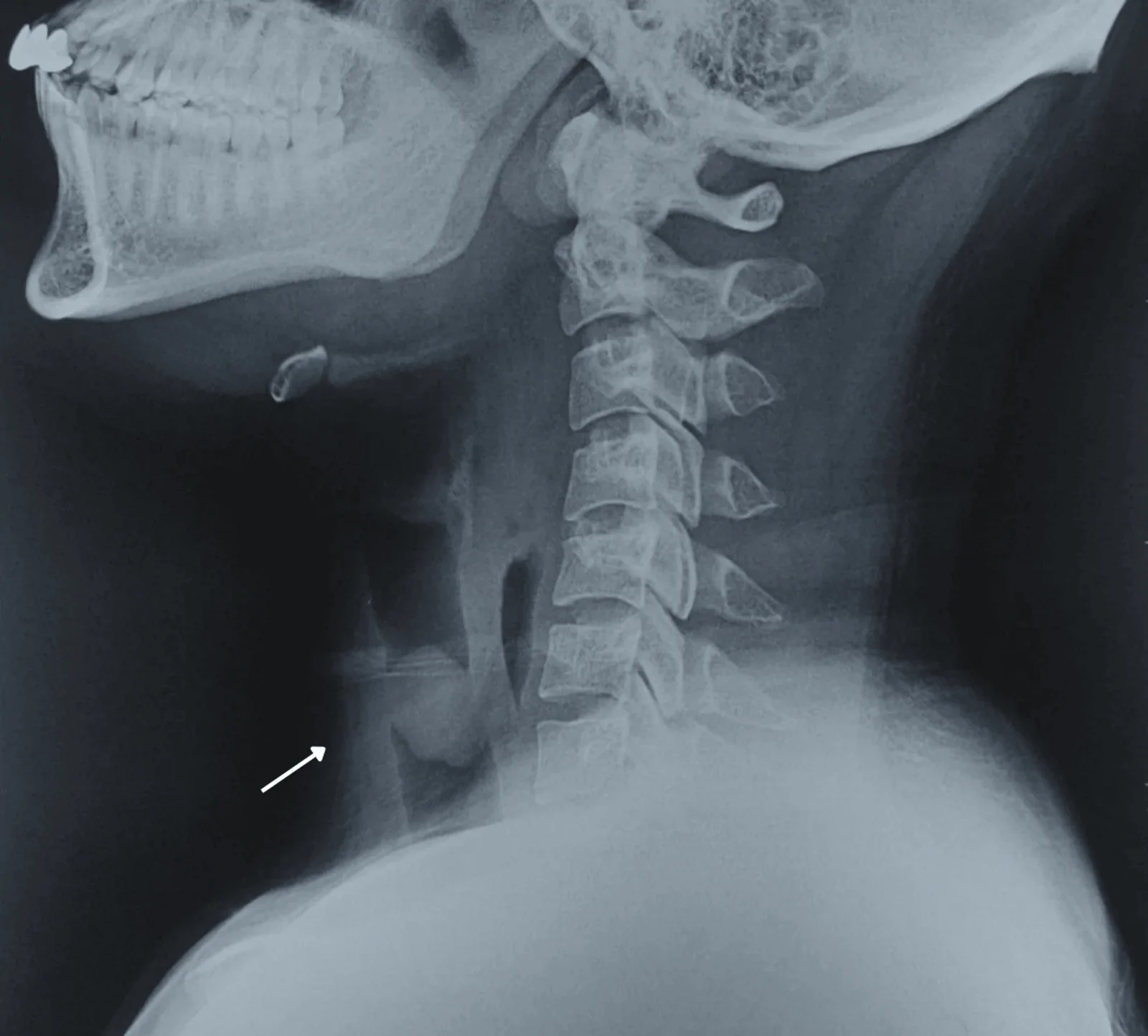
X-ray of the neck (lateral view) showing a soft-tissue opacity within the tracheal air column causing luminal narrowing at the C6–C7 level.

​Over the two weeks leading up to presentation, he developed progressively worsening dyspnea, prompting a consultation with the internal medicine department. A chest X-ray and a computed tomography (CT) scan of the chest were ordered to rule out pulmonary tuberculosis, which incidentally revealed a tracheal mass (Figure 1).

The patient was referred to the otolaryngology (ENT) department for surgical management. However, prior to his scheduled appointment, the patient experienced a sudden, acute exacerbation of breathlessness over a four-hour period, prompting an emergency department presentation in significant respiratory distress. On physical examination, audible inspiratory stridor was noted. His oxygen saturation was 92% on room air, and he was tachycardic with a heart rate of 118 beats per minute.

**Figure 2:**
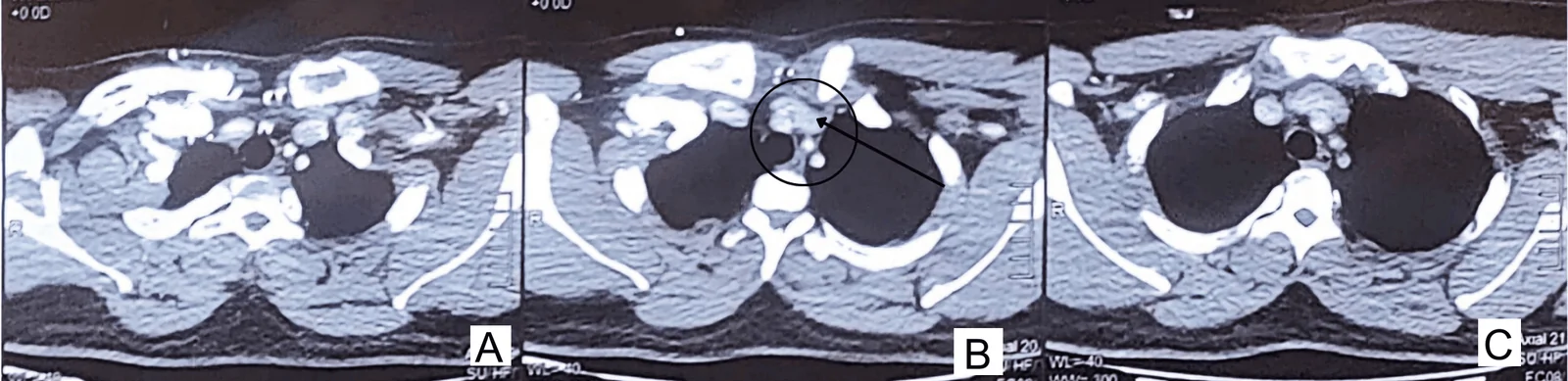
Axial contrast-enhanced CT image of the neck (A): At the level of C6 showing soft tissue thickening along the posterior tracheal wall with mild luminal narrowing; (B) At the level of C7 showing progressive intraluminal mass causing moderate airway compromise; (C): Inferior to C7 showing near-complete luminal obliteration by the posterior tracheal mass

​In view of impending airway compromise, emergency airway stabilization was immediately initiated, and the patient underwent an urgent tracheostomy. Following stabilization, a contrast-enhanced CT (CECT) of the neck and chest was performed. The CECT of the neck clearly delineated the tracheal mass (Figure 2). The CECT of the chest demonstrated a pneumomediastinum and a right-sided pneumothorax associated with bilateral dependent consolidation, likely secondary to the acute airway obstruction and emergency airway manipulation.

**Figure 3:**
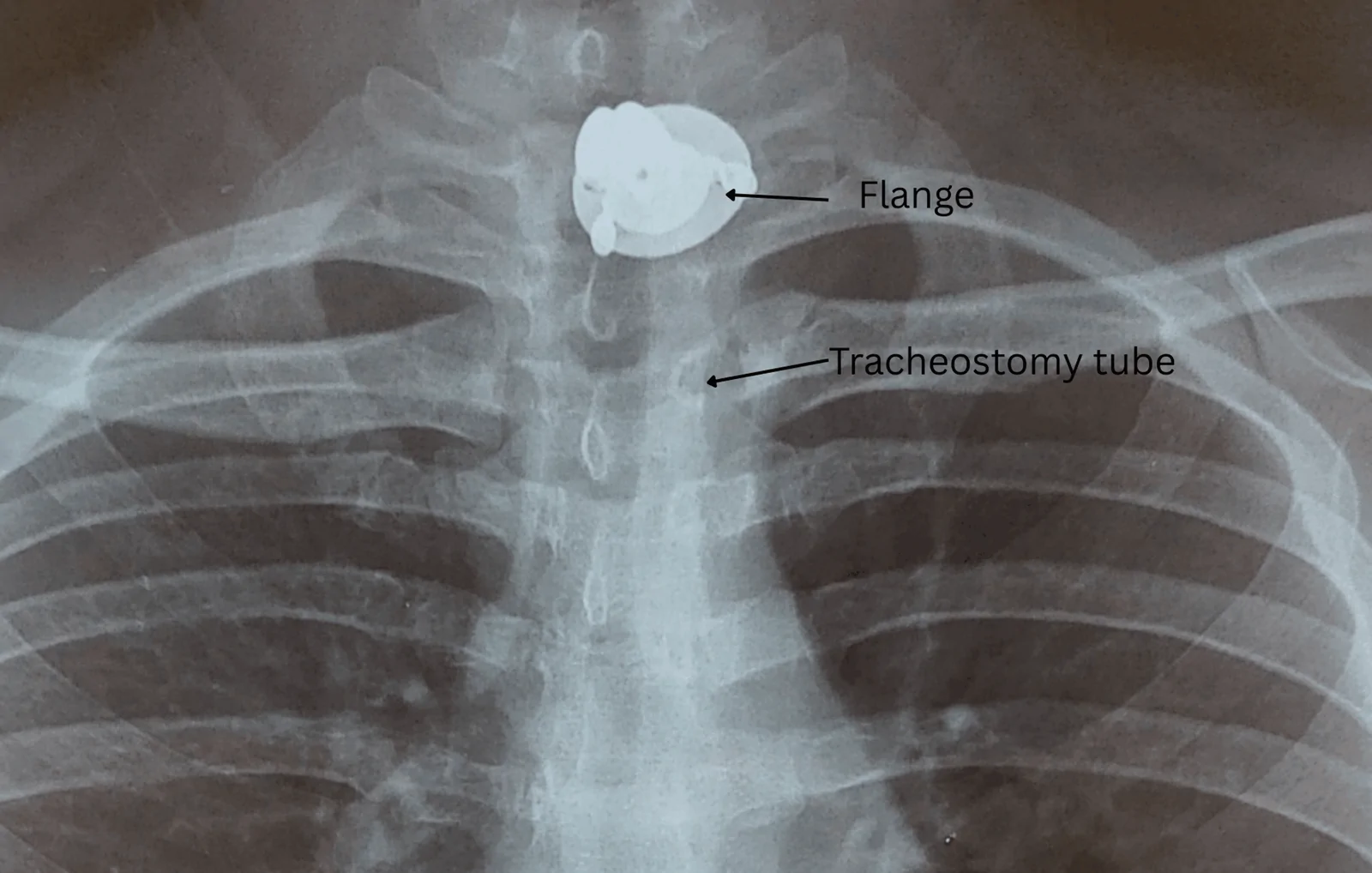
Chest X-ray (anterior view) done after tracheostomy confirming position

Chest X-ray done after tracheostomy to check the tube position is shown in Figure 3.

**Figure 4:**
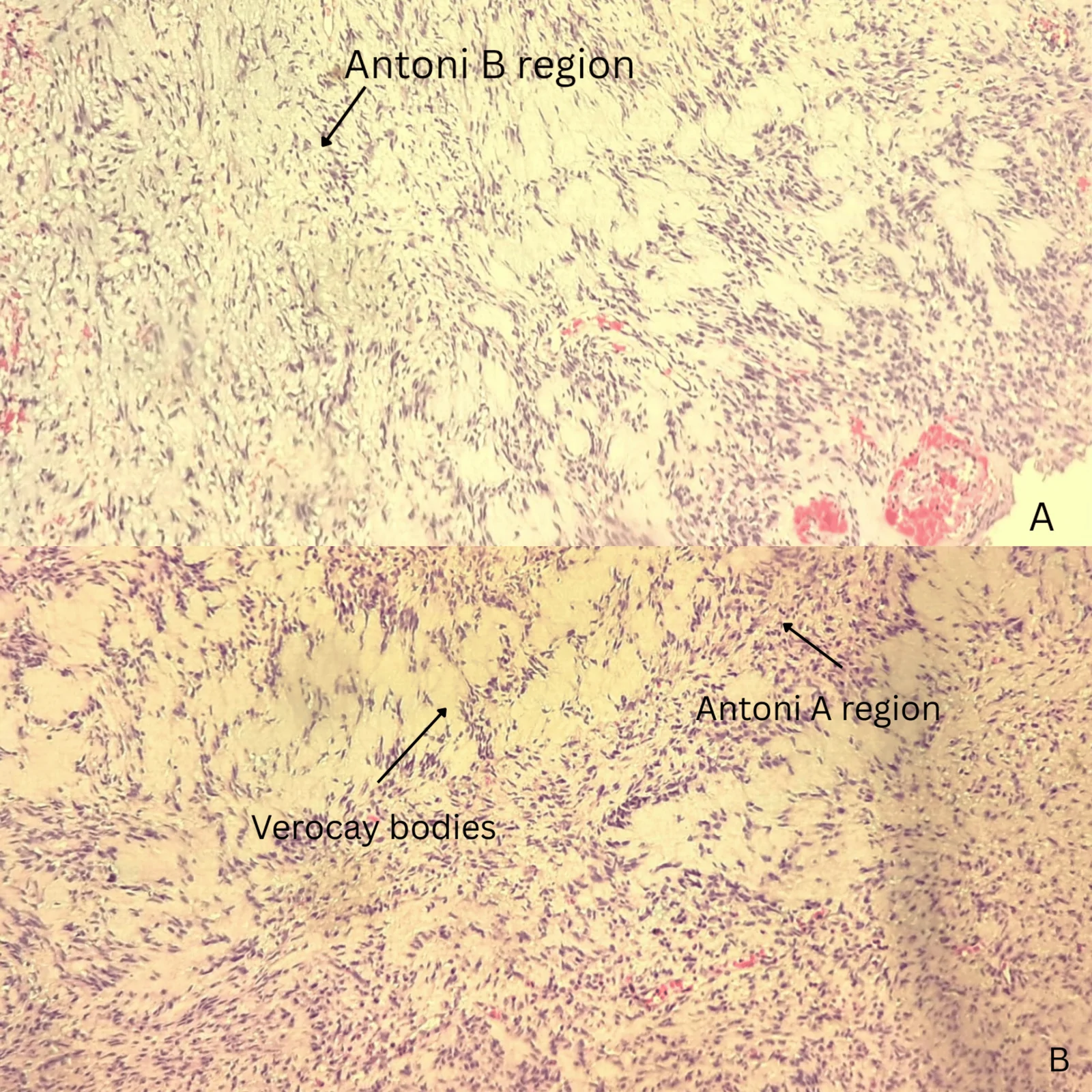
Histopathological features of tumor (A) Low power view showing spindle cell proliferation in storiform pattern and hypocellular (Antoni B) region, 100x; (B): Low power view showing hypercellular (Antoni A) area and Verocay bodies (nuclear palisading with central cellular zone), 100x.

Bronchoscopic evaluation revealed a smooth, well-defined polypoidal lesion arising from the tracheal wall, obstructing the airway lumen. The mass was pedunculated, pinkish in appearance, and located around the level of the third and fourth tracheal rings. The lesion was excised endoscopically. Gross examination revealed multiple grey-white soft tissue fragments measuring approximately 4 cc in aggregate. Histopathological examination showed spindle-shaped cells arranged in characteristic Antoni A and Antoni B patterns (Figure 4).

**Figure 5:**
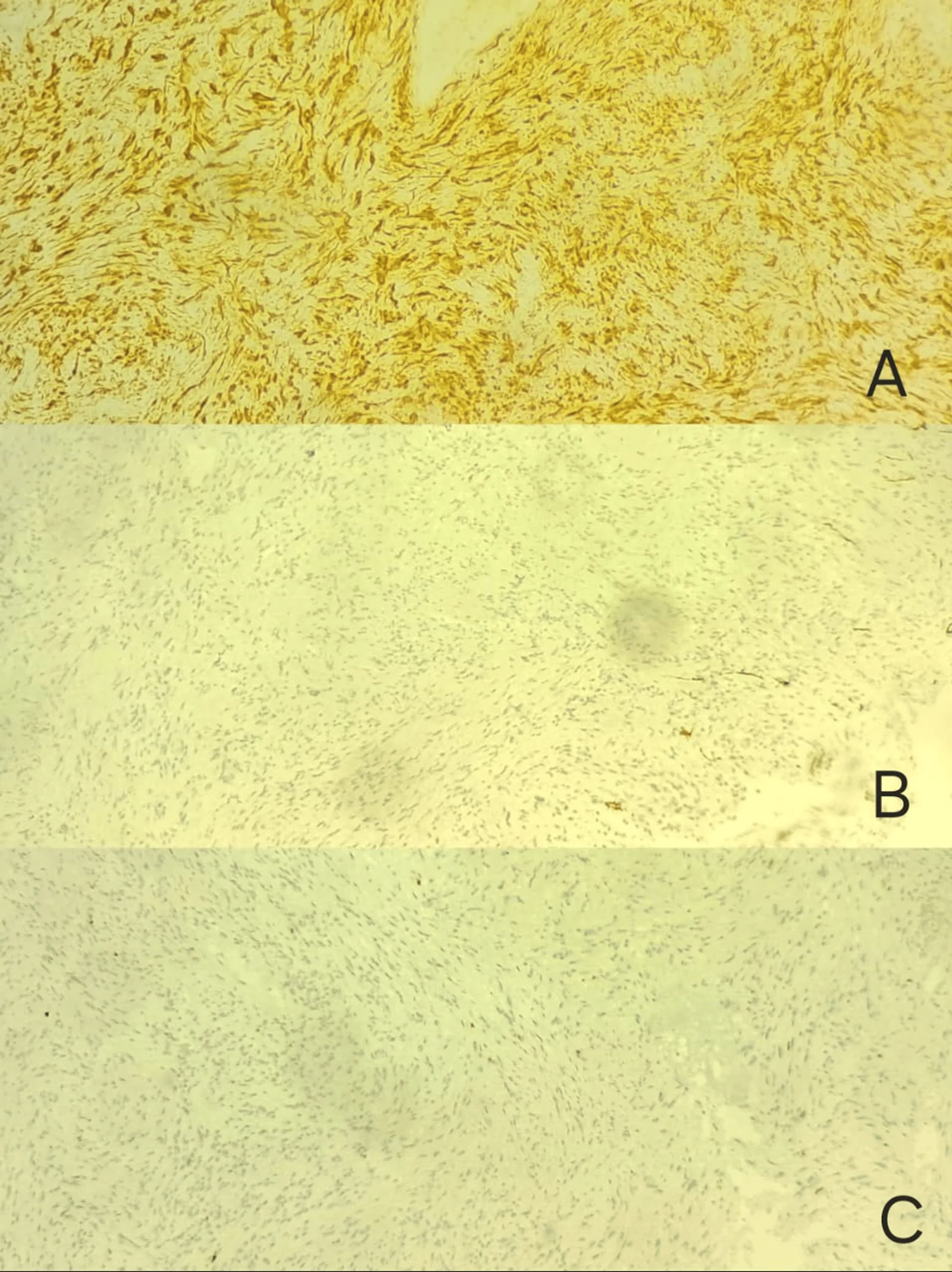
Immunohistochemical profile of the tumor (A) S100 immunostain demonstrating strong, diffuse cytoplasmic positivity in the spindle cells, confirming a neural crest/Schwann cell origin (100x); ​(B) Smooth Muscle Actin (SMA) immunostain showing negative expression in the tumor cells, ruling out a smooth muscle neoplasm (100x); (C) Ki-67 immunostain demonstrating a very low proliferation index, confirming the benign nature of the lesion (100x).

Figure 5A demonstrates diffuse S100 positivity, consistent with neural crest origin. In contrast, Figures 5B, 5C show the tumor is negative for SMA and Ki-67, respectively, helping us rule out smooth muscle origin and also cancerous nature (Table 1).

**Table 1:** Stains used and their results H&E: hematoxylin and eosin; SMA: smooth muscle actin

Stain	Result	Purpose
S100	Strong, diffuse positivity	Diagnostic hallmark for Schwannoma; confirms neural crest/ Schwann cell origin
H&E	Cellular (Antoni A) and hypocellular (Antoni B) areas	Microscopic evalualtion
SMA	Typically negative	To rule out smooth muscle tumor
Ki 67	Typically negative	Cellular proliferation index, confirms benign nature of schwannoma

The postoperative period was uneventful. The patient was discharged 25 days after tracheostomy in a stable condition. Objective assessment prior to discharge demonstrated stable oxygen saturation (SpO2 of 98% on room air). Laryngoscopic examination at this time confirmed the proper positioning of the tracheostomy tube in the anterior trachea, complete hemostasis, and a bulky trachealis muscle with no residual polyp or mass visualized. The patient was subsequently decannulated successfully. At the six-month postoperative follow-up, the patient remained completely asymptomatic, and surveillance bronchoscopy revealed a fully patent airway with no evidence of tumor recurrence.

## Discussion

Primary intratracheal schwannoma represents an exceedingly uncommon benign tumor derived from Schwann cells lining the neural sheath. Because of its slow growth and nonspecific respiratory manifestations, diagnosis is frequently delayed until significant airway narrowing occurs. Patients commonly present with cough, wheeze, dyspnea, or recurrent respiratory symptoms, often leading to an initial misdiagnosis of asthma or other obstructive airway disease. Other differential diagnoses for central airway masses include malignancies such as squamous cell carcinoma, mucoepidermoid carcinoma, and adenoid cystic carcinoma, as well as benign mesenchymal variants like leiomyomas [3-5]. In our case, the definitive diagnosis of a primary tracheal schwannoma was established via the unique immunohistochemical profile, which ruled out smooth muscle neoplasm and malignant differentials by demonstrating diffuse S100 positivity alongside negative SMA and Ki-67 immunostaining.

Recent literature highlights the diverse clinical presentations of this uncommon tumor. Mohamad et al. described a case presenting with subcutaneous emphysema and pneumomediastinum, emphasizing that airway obstruction may occasionally lead to unusual thoracic complications [2]. A recent narrative review by Alkhars et al. noted favorable outcomes with both endoscopic and surgical techniques when individualized to patient and tumor characteristics [6]. Thapa et al. reported primary tracheal schwannoma with extension to the thyroid gland, demonstrating that adjacent structure involvement, although rare, may complicate surgical planning [7]. Lorandi et al. similarly documented cervical schwannoma with tracheal invasion, expanding the spectrum of schwannoma-related airway compromise [1].

Radiologic imaging and bronchoscopy play a central role in diagnosis. CT typically demonstrates a well-defined intraluminal or mural tracheal lesion with associated airway narrowing. Bronchoscopic visualization allows direct assessment of tumor location, degree of obstruction, and tissue sampling when feasible. Definitive diagnosis relies on histopathological evaluation demonstrating spindle cell proliferation with Antoni A and Antoni B patterns, along with strong S100 immunoreactivity, confirming schwannoma [8].

Management depends on tumor size, location, extent, and the patient’s airway status. Open tracheal resection has traditionally been considered definitive therapy, particularly for large or infiltrative lesions. However, less invasive approaches are increasingly reported. Shen et al. demonstrated successful treatment with endoscopic resection, supporting bronchoscopic management in selected patients [9].

Our case was notable for rapidly progressive respiratory distress requiring emergency tracheostomy prior to definitive excision. Although schwannomas are benign and usually indolent, this case illustrates that acute airway compromise can occur when diagnosis is delayed, or luminal obstruction becomes critical. Prompt airway stabilization followed by complete excision resulted in excellent symptomatic recovery. This case reinforces the need to consider central airway pathology in patients with persistent cough, stridor, progressive dyspnea, or poor response to conventional asthma therapy. Early imaging and bronchoscopic evaluation are essential to avoid potentially life-threatening complications.

## Conclusions

Primary tracheal schwannoma is a rare benign airway tumor that may present with nonspecific respiratory symptoms, leading to delayed diagnosis. In patients with progressive dyspnea, wheeze, or stridor that is unresponsive to conventional therapy, central airway obstruction should be considered. Early imaging and bronchoscopic evaluation are essential for prompt diagnosis and prevention of life-threatening airway compromise. Definitive management with endoscopic or surgical resection generally results in excellent outcomes. This case highlights the importance of maintaining a high index of suspicion for uncommon tracheal lesions in young patients presenting with acute respiratory distress.
